# The Elements of Success

**Published:** 1887-05

**Authors:** W. C. Barrett


### THE ELEMENTS OF SUCCESS.

BY DR. W. C. BARRETT.

AS READ BEFORE THE CENTRAL DENTAL ASSOCIATION OF NORTHERN NEW JERSEY.

You have asked me to address you upon a subject that permits a wide range of thought. What are the elements of success, either in dental practice or in any other vocation ? Are they natural or acquired ? Does dentistry demand different qualifications in its exemplars from other callings ? These are questions that may well engage our most earnest attention as dentists.

Before attempting an analysis of the means and methods by which the summit of professional ambition is attained, it will be well for us to have a fair understanding of what is meant by success in practice. Different men have widely variant ideas as to the end which it is their ambition to attain. That which would content one might fall far short of the desires of another. The bent and tone of the human mind differs with the individual, and the pleasure of one is the harassment of another. Some find their greatest gratification in labor, while others long for nothing so much as opportunity to gratify their indolence. One man views everything from a pecuniary stand-point, and considers the acquisition of wealth the sole measure of success, while others make the obtaining of knowledge their only good. Some men are most highly desirous of station and influence; of power and prerogative. As the one views everything from a financial stand-point, so the other looks at all matters with reference to their ability to pander to his love for notoriety. It is hard to say which object is of itself the more despicable. There are a few—I fear but too few—who are entirely philanthropic in their aims; whose honest desire is to do the most good possible in the world, and to do that in the most unostentatious way. All honor to such. It is a pity that they are so likely to be lonesome.

I believe that the majority of honest men in our profession have mixed motives. They desire success that it may bring them a competency, and this wish is thoroughly praiseworthy. They hope to succeed that they may become known, and be respected by their fellows, and this too is a high ambition. They desire to be useful, both because of their real humanity and because of the knowledge that it is only the useful man who is thoroughly respected. Here, then, we have three motives that actuate honorable men; the desire for wealth, for reputation, and to be of use to their fellow-men. How shall these things be secured ?

The foundation for success in life must be laid by the parents of the coming man. Before the child is old enough to comprehend what is best, or to mark out a line of life, his training should be well under way. In this day of enlightenment the man who is without a fair English education is heavily handicapped in the race of life. This education should commence in early childhood. Not only a training in the schools, but the physical and moral culture should be begun early. One-fourth of man's three score and ten years should be spent in preparation for the active duties of life.

Not before the age of eighteen is the young man's judgment enough matured, or his experience sufficiently varied to enable him safely to mark out a path for the future and to select his vocation. Probably nine-tenths of us are governed by circumstances and environments in our choice of a means of livelihood, and that is why so few of us are really successful. The round pegs get in the square holes, and it is no wonder that they do not fit. To very few of us is it vouchsafed that we shall be found in exactly the right place when the opportunity of life comes. The majority, if not the whole of us, are endowed with certain qualities that, if the proper occasion arrives, will carry us on to fortune. General Grant waited many years in darkness and doubt before he found the opportunity to display his abilities. Did you ever think of the possibilities that are wasted; of the heroic qualities that never have an opportunity to exhibit themselves? The poet Gray, in musing in a country church-yard, saw this, and beautifully expresses it—

> "Perhaps in this neglected spot is laid,
> Some heart once pregnant with celestial fire;
> Hands, that the rod of empire might have swayed,
> And waked to ecstasy the living lyre."

You that are parents should not forget that the success or failure of the child given to your training is largely dependent upon the way in which you perform that duty. The temptation to moralize in this connection is very great, but I must recollect that you probably expect from me something of a practical character. You desire that I shall say something that will the better enable you as dentists to reach the goal of your ambition, and I must confine myself within professional limits.

The successful dentist, then, must be an educated man, and must possess a disciplined mind. Dentistry aspires to rank with the learned professions. The accepted dentist is no longer an empiric, a mere artisan, a workman. He is entrusted with grave responsibilities, and he must be qualified for his task, or he is unfit for his vocation. Within a few years—easily within the recollection of even young practitioners—the law has, in most of the important States, prescribed what shall be his qualifications. It has very properly announced that the ignorant charlatan shall not undertake the performance of duties for which he has made no effort to fit himself. This has elevated our status immeasurably. New York was one of the early States to secure proper legislation, and, with others, I labored for years before I saw the attainment of my desires. I can look back upon the status of dentistry in my own State, before the passage of the act, and it seems to me amazing, the change that has been wrought in the estimation of the public. So prejudiced were our people, that we were obliged to undergo a probation of years under a law that simply recognized us, before we dared to ask the legislature to place any restrictions upon those who were desirous to enter our field. It was imperative that we should first educate the laity to an appreciation of dentistry. That has been done, and now we have a law that is not only sustained by jurists, but by the sentiment of the people as well, and we are secure in our acceptation. This appreciation on the part of the public is constantly augmenting, and the time is at hand when intelligent people everywhere will refuse to acknowledge or patronize the dentist who has not the diploma of some reputable college.

It is absolutely essential, then, that the dentist of the future should possess a well-earned diploma. We are now in the transition stage of professional development, and I am aware that many are in the practice of dentistry who have not had scholastic advantages. But that does not mean that they are ignorant, or if so, that they need remain in that condition. There are abundant opportunities for study, and the man who is imbued with a proper spirit will not be content while there is a single principle unattained.

Having obtained a professional education and entered upon practice, the most critical period in the life of a young dentist commences. His professional habits are yet to be formed, and his feet are to enter upon the road that they will in all probability follow to the end. He is apt to be puffed up with a feeling of his importance. Too often he imagines that his professional education is ended, when in fact it should be but just begun. He is often inclined to rely upon what he has already accomplished, and to trust to his genius, his talent, for the future. Genius! if he have it, the chances are a hundred to one that it will prove a curse to him. The possession, or the imaginary possession, of great ability has ruined more promising young men than any other one thing. There is no such thing as intuitive genius, save the capacity for work. An ounce of industry is worth a ton of talent in any market. It is the man of patient, plodding perseverance who soonest reaches the goal. It is the man who always has a book at his elbow, who is constantly endeavoring to master the underlying principles of his profession, who is never boastful of his own attainments and thus shutting up the avenues of information, but who is rather desirous of obtaining knowledge from any source—he is the one who succeeds. When a young man enters my office, or when I encounter him at a society meeting or elsewhere, and he begins to tell me of his great success in practice, of his unfailing remedies and methods, and of the great numbers who flock to obtain the benefits of his matchless skill, I do not need the eye of prescience to foretell what his future will be. He is instantly weighed in my mental balance and invariably found wanting. He knows too much to receive information from another, and will surely be overwhelmed by his flood of egotism.

But when a young man comes to me and candidly admits that he sometimes meets with difficulties, when he desires to know what should be done in such or such an emergency, how I perform this operation and why I prescribe certain remedies, when he seems possessed with a thirst for professional knowledge and a determination to master every method of manipulation, I know that he will go on acquiring intelligence until the sum of it all will place him in the front rank. Look at the men of acknowledged skill and watch their conduct in the office of a brother dentist. I have had such men visit me, men whom I would be proud to acknowledge as my masters, and I was fairly embarrassed at the attitude of pupilage in which they placed themselves. My mind's eye now rests on one of whom almost any one of us would be glad to learn, and when he entered my office there was not an excavator which he did not carefully examine. Not for the purpose of criticism, but to see what he could learn. There was not an implement which was at all peculiar to me and my methods (we all have our own personal specialties) which he did not exhaustively examine, and of which he did not demand a demonstration. I could see that he was mentally comparing it with his own instruments and methods, and inquiring if he could not better his practice by adopting some of my ways. Had he been an entire stranger to me, I could have read his professional standing by the incisiveness of his questions and his consuming thirst for that which was the best. And what this man was in my office, that he is wherever he goes.

The really earnest man will find instruction everywhere. Some of the most valuable hints that I have obtained I have found in the most unlikely places. I remember getting snowed in on a train, at a small country village, and much against my will spending a day there. There was but one dentist in the place, and evidently he was poorly appreciated. Some of his methods were crude in the extreme, and he was, to all appearance, steeped in poverty to the lips. But I have seldom spent a more profitable day than the one passed in those apparently unpromising rooms. Poor as he was, he was a subscriber to all the best journals, and it was fairly amusing to witness the ingenuity with which he had succeeded in forcing make-shifts of his own devising to supply the place of the expensive apparatus of which he had read. He had no patent saliva-ejector, but he made a piece of soft linen (an old napkin) act as a capital siphon. His hot-air syringe was a common rubber bulb, to which he had attached a piece of brass tubing. Exhausting this, and holding the point over the flame of his alcohol lamp, it was speedily filled with air as hot as he desired it. His rubber dam was carefully economized, and when it leaked, a long spiral of bibulous paper siphoned out the moisture as fast as it entered. There were many other little devices that I saw, which have proved useful to me ever since.

I have indulged in this discursion that I might illustrate my assertion that genius is but another name for industry. The young dentist who has a successful career before him is a man of industry and determination.

There is another quality that the young man just starting in a professional life must possess, and that is an enthusiastic love for his calling. If he views it simply as a means of subsistence, as drudgery to which his unfortunate lot in life has doomed him, failure is certain. He must honestly believe that his is one of the noblest callings which man is privileged to follow. He must be ready to worship the good Providence that has graciously permitted him the high privilege of being a dentist. There must be no drudgery in it to him. He must love his work better than play. His office, next to his home, must be the dearest spot on earth to him. He must possess an absolute affection for his chair and his instruments. He must prefer the society of intelligent dentists above that of any other possible class of men. His successes in practice must be triumphs to him, and he must value a piece of work well done above the honors of the unprofessional world. The time for closing his office must come as a surprise, and the hours spent in his professional engagements must fly as upon the wings of the wind. His heart and mind must be so engrossed in his labor or his study that the days shall seem as hours. When a young man takes hold of his life's work in this way, it is no longer labor. That is not wearisome which we love to do. That is no drudgery in which the heart is engaged. The hours are long and heavy when our bodies are chained to uncongenial toil; when the mind finds its delight where the body is not.

Nor is it impossible for even the dullest laggard to obtain this gladness in his task. Every one can school himself to enthusiasm in his profession if he will but give to it his whole attention; if he will let nothing distract his mind from his business during business hours.

This habit of study must be acquired if success is to be attained. The mind is so constituted that it will find enjoyment in that to which it is constantly directed. A habit of reading is as easily attained as the habit of smoking or chewing tobacco, to which so many dedicate long years, and it is quite as difficult to abandon when once acquired. One can attain a habit of reading scientific and professional books with as much facility as he can the perusal of works of fiction. If his affections are with his business, he will delight to read of that business, and that reading is a necessity for the successful man. The progress of dentistry is so rapid, and the advancement is so marked, that he who sits down for but a single day will find himself left behind. We are moving at a quick march, and only the active can keep up. When I return to my office after a week's absence, I always find an arrearage that must be brought up if I would keep abreast the advance of thought. I dare not let a single number of any of our journals go unread, for if I do I am sure to have dropped a stitch, and soon there will be something in dentistry which I cannot comprehend, because of that interregnum. Dental practice has no place for the lazy man. He will certainly be shoved to the wall, and have the mortification to find himself at the tail-end of the procession.

The young man who aims at success must exercise some judgment in pursuing his studies. He should study principles, and the comprehension of general law. There is no one so contemptible as he who is continually seeking after recipes, and specifics, and cure-alls, and infallible methods. A young man lately wrote me asking for an unfailing cure for periostitis. The ignorant dolt did not know what periostitis is. His question told me that. A remedy that should cure a disorder that may have a hundred causes, and require as many different methods of treatment! The very idea is an absurdity. He was one of those specific-hunters; one of the men who treat diseases by recipes and unbending rules. No man is qualified to properly attend a case of dental periosteal inflammation until he has obtained a knowledge of what the condition is, and by what circumstances it is modified.

The successful dentist will study to comprehend principles. He will endeavor to go to the root of the matter, and find out what the exact pathological condition is. Then he will study the therapeutic qualities of drugs, and thus become qualified to properly dispense them. When I see a young man filling a note-book with formulas, and recipes, and arbitrary prescriptions, I think he has mistaken his calling. He should have been a carpenter, or a mason, and then he could always have moved by square and compass. A profession differs from a trade in this: that whereas the one is governed by principles and fundamental truths, and its practice is the application of knowledge and judgment, the other is the mere following of unvarying rules, and the working by a mathematical law which makes no demand upon the judgment or intelligence. No two cases of the same disease in different persons should be treated exactly alike; but the blacksmith makes all horseshoes in the same manner. Painting is an art; photography is a trade. The one is an affair of the judgment, the intelligence; the other is prosecuted by rules and recipes. Study, then, must be intelligently directed, and not devoted to the mere accumulation of dry facts.

You will not hear the successful dentist, or he who has in him the elements of success, complaining that he has no time for study. As soon would you hear him lamenting that he had no opportunity to breathe, for study to the really successful man is as essential as breathing. Of all the miserable quibbles to which men will resort as an excuse for duty unperformed, that of "no time" is the most contemptible. Who are these men with no leisure? Are they the dentists of large practice? No, for whenever and wherever you meet a really successful practitioner you will find him a reading man; one who has pleanty of time to keep abreast the advance of thought. It is this very study, this constant comparison of the views and methods of others, that makes him successful. The men whose names are best known in our literature are those who have large private practices.

I might make out for you a list of the names best known in dentistry, and you would find all of them men with large private practices, who yet find time to read their professional literature, to lecture in colleges, to make exhaustive experiments, and to write frequent articles for the journals. There is no complaint from them of a lack of time for study, and they are the successful men of our profession. They are studious men because that is their professional life, and through their studies they have conquered success.

It is the men of small caliber who have "no time." It is the dentist with a practice of a thousand dollars a year who cannot find leisure for self-improvement; these are the ones who, if they subscribe for a professional journal at all, must have one which contains no long articles. It must be made up of extracts and pithy sayings; of recipes and specifics. These men have "no time" for the study of complete disquisitions upon any subject. One journal is quite enough for them. They cannot possibly find the time to read two.

The successful man finds plenty of time for reading. Not a book on dental subjects is issued that he does not carefully study. It is this that has made him what he is. And yet, from the men at the very tail end of dentistry we frequently hear the complaint, "Oh, I cannot subscribe for this or that journal. I have not the time to read those which I now take." At the tail such men are now, and at the tail they will always be found.

Sometimes we hear the excuse from dentists that it costs too much to take dental journals, and to buy dental books. They really cannot afford it. Do you remember the words of the author of the Biblical Proverbs? "There is that scattereth and yet increaseth; and there is that withholdeth more than is meet, but it tendeth to poverty." The dentist who denies himself the proper instruments with which to do his work will starve his practice, and his patients will leave him for the one who has better facilities, and who will, therefore, be enabled to give better satisfaction. The practitioner who shuts the avenues of professional information is a worse economist than the other, for he deliberately robs his patients of that which they have a right to expect, and denies them that which he should be competent to furnish them.

The successful dentist is a constant attendant upon society meetings. It is there he meets his brother practitioners, measures himself by their standard and is stimulated to higher endeavors. He hears the methods of others discussed, and learns the best. All the new ideas are introduced, and he reaps the benefit of the years of experience of the best men in his profession. Quacks never attend society meetings, but I challenge you to produce the name of one man who has won even a moderate reputation, and who is not a frequent attendant where dentists are gathered together. I defy you to name one dentist who has been an attendant upon society meetings, and who has tried to intelligently follow the papers and discussions, who will not solemnly declare that he has been directly reimbursed many times over for the expenditure of time and money that these meetings demanded. Success in practice cannot be attained without the interchange of ideas and methods that is only secured at professional assemblies. You will find it a direct result of your attendance of society meetings that your fees will become larger, through your ability to do better work. It is not dishonest to obtain heavy fees, if only your work is commensurate with your charges. Men can get just what they are worth, for there are many patients who desire the best, and who are ready and willing to pay the highest fees for the most valuable services. The faithful dentist strives to excel by improving every chance to obtain information, and pecuniary success follows as the night the day.

The successful dentist is an honest man. He not only strives to be the best informed, but he sedulously studies to give to every patient the benefit of his whole skill. I am well aware that there are times when the brain and hand are tired, and the temptation is almost irresistible to take the easiest road for one's self, regardless of what is best for the one who has entrusted himself to our care. A cavity is difficult of access, and labor for the overstrained muscles might be saved by careless excavating and a hastily inserted filling. Just at the critical point in an operation a leak in the rubber dam is discovered, which has flooded a partially filled tooth. It would be so easy to finish with a plastic make-shift that tired nature protests against the honest course. Or, at the close of a protracted operation it would be so much less labor to end the filling when it is flush with the margins, instead of continuing the work until the gold is carefully built over and around the frail, infirm walls, thoroughly protecting them from injury, that we are tempted to stop midway. This is not the road to success. If the dentist is weak enough to yield to evil thoughts and suggestions, he may be sure that the fault will return to plague him when it has long been forgotten. If he once gives way to the allurements of his evil nature, the next lapse of integrity will be easier, and the down-hill road entered upon it will be but a little time before he will find himself a confirmed, slovenly operator, and his reputation for honest work will be irretrievably ruined. His patient pays him for his best services, and the dentist who does not in every case give all that he constructively contracts to give, is a cheat and a fraud. He has sold a thing which he refuses to deliver, and he may be sure that his patient will sooner or later find him out, and he will reap the reward of his dishonor in a diminished practice and a tainted reputation.

The successful dentist is a man who is neat in his personal habits. Refined people will not twice visit the man whose finger nails are in mourning for his departed cleanliness, nor whose person is reeking with the fumes of the putrefying exuviæ from his glandular system. If he be a sloven in dress, the inference is a fair one that he will be the same in his work. He will not go to the chair wearing the same coat that he wears about his other avocations. He will carefully, and even ostentatiously, wash his hands before going to a new patient. No one wishes the saliva of another introduced into his mouth upon the unwashed fingers of the dentist.

Not only will he be cleanly in his person, but all his instruments will be bright and shining. Not a speck of dirt will be found upon the points of his excavators, the remaining filthiness of his last operation. His office furniture will be carefully brushed, and his carpets scrupulously swept. His office fixtures may be plain, but they will at least be in perfect order. Above all, his spittoon will be thoroughly washed after every time of using. The least suspicion of blood about it will be quite enough to disgust refined people, and send them away to return no more. I once received a lesson that is not yet forgotten, when I was told by a patient that a lady of an excellent family, and one which I was very anxious to secure, had visited me at the recommendation of the relator, with the view of placing herself under my care, but that the sight of a bloody napkin, which my office girl had carelessly left in an exposed place, but which had not met my eye, caused her to leave me in disgust. You may be sure that I have kept my eyes open for such offensive exhibitions since that time.

The successful dentist will be careful and fastidious in his talk. He will not introduce offensive subjects in conversation, and he will strive to clothe his thoughts in well selected words. He will not be affected or priggish, but he will strive to speak grammatically. Educated persons will not seek the society of those who are continually offending propriety by an exhibition of illiteracy and boorishness. Above all, the successful dentist will never indulge in vulgarity, even though his patient may be coarse and unrefined. Every one expects that a professional man will be grave and decent in his professional demeanor. The gross remark and the indelicate story, if indulged in at all, will be reserved for the time when he is not engaged in professional duties.

The successful dentist will be tender and sympathizing in his demeanor to those who are necessarily made to suffer at his hands. It is but the natural consequence that he should become inured to scenes of woe and pain, but he will be careful to avoid exhibiting it. It is his daily duty to cause suffering, and he will become hardened to signs of agony which would move the unprofessional eye to tears. But though he does not feel the woes of his patient, he must not fail to express the tenderest sympathy. He will carefully avoid and faithfully guard against the natural hardening of his feelings, and will strive, as far as possible, to keep his sympathies alert. But if, unhappily, he becomes so habituated to scenes of suffering that his tenderness is exhausted, his patients will not find it out. Nothing so engages the confidence of the weaker part of his clientele as to receive the impression that the operator is tenderly alive to all the pain which he unavoidably causes. He will show an earnest, careful, anxious interest in the welfare of his patients, and give them reason to believe that he will care for them as tenderly as though he were a brother. In making examinations, he will assure them that he will be as tender as though it were his own flesh and blood that was to suffer by any carelessness, and he will then so conduct his manipulations as to preserve the confidence thus gained.

The world is crowded with the suffering, and no dentist can, without unduly sacrificing himself, allow his sympathies to be too deeply engaged in any case, but his manner must never betray him. He must depend chiefly, of course, upon his actual skill, but he will find that not one in twenty is competent to judge of his technical ability in any other way than by experience, and so his early patients must largely be won by his manner. The majority will be governed by the care and devotion that he exhibits, and will form their opinion of him and measure his services by the details of routine transactions. But the dentist will be careful not to let this degenerate into a semblance of weakness and indecision. With all his gentleness he will be firm and determined, and will endeavor to sustain the weak by his exhibition of mental strength. His will be the hand of iron, covered and masked by the glove of kid.

The dentist who proposes to attain to a good practice will be careful how he speaks of his brother practitioners. He will build upon his own foundation, and not by tearing down the reputation of another. The temptation to disparage a rival is sometimes very great, but let me ask if you ever knew any one to reach eminence who habitually indulged in this habit? Patients soon learn to distrust one who is continually attacking the reputation of his brethren, and set him down as an envious, malicious, untrustworthy maligner. There is a love of fair play implanted in the breast of almost every one, and if an absent and defenceless brother be attacked, the sympathies of a right feeling patient are sure to be enlisted in his behalf, and the chances are that the backbiter will lose and the maligned dentist gain a patient. If a practitioner is on bad terms with his professional brethren, it argues that there is something wrong in himself. Either he feels his inferiority and is envious of the superior attainments of his neighbor, or his own disposition is malicious, and he would wontonly inflict injury on others. In either case the impression made upon his patient is bad, and the offending dentist will himself be the greatest sufferer from his own indiscretion.

The successful dentist will be a good business man, and will not allow his bills to be too long unpresented. The best time to remind the debtor of his indebtedness is when it is fresh in his mind, and before it shall have passed from memory. More disputes are engendered by a long delay in presenting bills than from any other business cause. As soon as a series of operations is concluded, a statement will be sent, that no future misunderstandings may occur. In no case will this duty be delayed longer than the first of the month succeeding that in which the work was done. If immediate payment be not urgently desired this fact will be conveyed to the debtor, and he will be made to know that the statement is rendered that errors, if they shall have occurred, may be rectified before the particulars shall have been forgotten. The professional man will not be the less respected for asking prompt payment. He has rendered faithful service, and now he is entitled to his just reward.

There are many other qualities which the successful dentist will possess, but they may all be summed up in saying that he will be an earnest, industrious, studious, active gentleman, possessed of self-respect and respecting the feelings and right of others. He will so conduct himself that he shall win the honest regard of good men and women. He will be the true professional man in all the relations of his life, remembering that there is something higher, nobler, more worthy the best exertions of man, than the mere acquisition of money or the gaining of that notoriety which so many consider equivalent to an honest, well-won and modestly supported reputation.

